# Retinal microvascular impairment in COVID‐19 patients: A meta‐analysis

**DOI:** 10.1002/iid3.619

**Published:** 2022-05-11

**Authors:** Suzhen Wang, Jie Wang, Jianbin Hu, Ningli Wang

**Affiliations:** ^1^ Eye School Chengdu University of Traditional Chinese Medicine Chengdu China; ^2^ Aier Eye Hospital (East of Chengdu) Chengdu China

**Keywords:** COVID‐19, optical coherence tomography angiography, retinal microvascular, SARS‐CoV‐2, vessel density

## Abstract

**Background:**

The coronavirus disease 2019 (COVID‐19), caused by severe acute respiratory syndrome coronavirus 2 (SARS‐CoV‐2), has led to a global pandemic in an unprecedented time frame. Systemic vascular involvement in COVID‐19 has been identified, and SARS‐CoV‐2 has also been found to cause multiple organ ischemia and posterior ocular segment disease in mammals, raising concerns about the human retinal microvascular involvement in SARS‐CoV‐2.

**Objective:**

To objectively assess the presence of retinal microvascular impairment in COVID‐19 patients by optical coherence tomography angiography (OCTA), so as to facilitate the clinical system management of COVID‐19 patients.

**Methods:**

We searched PubMed, Cochrane Library, EMBASE, Ovid, CBM to collect eligible studies. The main outcomes included the vessel density (VD), area or perimeter of foveal avascular zone (FAZ), central foveal thickness (CFT), subfoveal choroidal thickness (SCT) in our meta‐analysis.

**Results:**

We eventually included five studies with a total of 401 participants. Our meta‐analysis showed that nonacute infectious COVID‐19 or post‐COVID‐19 patients presented significantly lower foveal VD of deep capillary plexus (WMD = −4.22, 95% CI [−8.00, −0.43]) and thinner SCT (WMD = −10.33, 95% CI [−19.08, −1.57]) than healthy controls. The foveal VD and parafoveal VD of superficial capillary plexus, parafoveal VD of deep capillary plexus, CFT, area, and perimeter of FAZ showed no significant differences between the groups.

**Conclusion:**

The patients of nonacute infectious COVID‐19 or post‐COVID‐19 displayed alterations in the retinal microvasculature and choroidal vessels, including a significantly lower foveal VD in deep capillary plexus and thinner SCT. The impairment may be a medium to long‐term process. Close ophthalmic surveillance is necessary for COVID‐19 patients or post‐COVID‐19 patients.

## Graphical abstract

Our meta‐analysis showed that patients of nonacute infectious COVID‐19 or post‐COVID‐19 presented significantly lower foveal vessel density of deep capillary plexus and thinner subfoveal choroidal thickness than healthy controls. The impairment of SARS‐CoV‐2 to retinal microvessels and choroidal vessels maybe a medium to long‐term process. Close ophthalmic surveillance is necessary for COVID‐19 patients or post‐COVID‐19 patients.

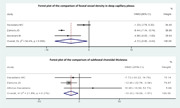

## 1 INTRODUCTION

The coronavirus disease 2019 (COVID‐19), caused by severe acute respiratory syndrome coronavirus 2 (SARS‐CoV‐2), has led to a global pandemic in an unprecedented time frame.^1^ COVID‐19 is a highly contagious disease that causes severe respiratory, immune, and other organ systems dysfunction, leading to severe morbidity and mortality.^2^ Studies have shown that COVID‐19 patients manifest as cardiovascular, neurological, hematological, cutaneous, and taste, gastrointestinal symptoms in addition to the usual fever and respiratory symptoms.^3^ Various ophthalmic manifestations of SARS‐CoV‐2 infection have also been reported, including anterior segment involvement such as conjunctivitis, keratitis; and retinal microangiopathy.^4^, ^5^, ^6^

It is reported that the cell surface enzyme protein angiotensin‐converting enzyme 2 (ACE2) is one of the major actors for SARS‐CoV‐2 entry into host cells.^7^ In humans, ACE2 has been observed in the central nervous system, blood vessels, lungs, nose, immune system, conjunctiva, cornea, and retina.^8^, ^9^, ^10^, ^11^ A study conducted by Casagrande et al.^12^ showed that SARS‐CoV‐2 virus RNA was detected in the retinas of 3 out of 14 eyes of deceased COVID‐19 patients. In addition, coronaviruses cause a variety of ocular infections in animals, including retinal disease, which has been demonstrated in feline and murine models.^13^ Microvascular endothelial injury and cytokine oversecretion are also considered to be the key factors leading to multiple organ failure in patients with severe COVID‐19.^14^ Vascular endothelial cells play an important role in controlling vascular tension and protecting the blood‐retinal barrier through their active paracrine, endocrine, and autocrine functions. Despite recent data showing retinal microvascular impairment in COVID‐19 patients, the validity of these studies has been controversial.^15^, ^16^ In the context of the current SARS‐CoV‐2 pandemic, the study of retinal vascular changes in SARS‐CoV‐2 is of great significance for follow‐up and identification of possible short‐ and long‐term sequelae in COVID‐19 patients.

The retina is a relatively convenient organ that can be used to assess and quantify these microvascular changes by directly examining blood vessels. The optical coherence tomography angiography (OCTA) is a novel, noninvasive three‐dimensional retinal angiography technique that can quantitatively evaluate retinal blood vessels, making it a unique advantage in the study of COVID‐19‐related microvascular impairment.^17^ The purpose of our meta‐analysis was to objectively assess the presence of retinal microvascular impairment in COVID‐19 patients by OCTA, so as to facilitate the clinical system management of COVID‐19 patients.

## 2 METHODS

### 2.1 Search strategy

We searched PubMed, Ovid, Cochrane Library, EMBASE, CBM databases for relevant literatures without language restrictions from December 2019 to May 2021, using medical subject headings and free words combined with COVID‐19, ocular, eye, retina, retinal vessels, vessels, microvessels, OCTA. We also carefully sifted the reference lists of published reviews to identify applicable literature.

### 2.2 Eligibility criteria

We selected the literature based on the following criteria: (1) patients with confirmed diagnosis of COVID‐19, or patients who have recovered from COVID‐19, (2) comparison of COVID‐19 or post‐COVID‐19 patients with healthy controls without COVID‐19 infection, (3) the type of literature included was observational study, cross‐sectional study, retrospective study, randomized clinical study, and cohort study, (4) at least one interesting outcome was reported in the literature, including the macular vessel density (VD), area or perimeter of foveal avascular zone (FAZ), central foveal thickness (CFT), subfoveal choroidal thickness (SCT). Conference abstracts, letters, duplicate publications, case reports, reviews, and studies with incomplete data were excluded.

### 2.3 Data extraction and quality assessment

Two researchers independently screened titles and abstracts of the retrieved studies, and then carefully read the full article to determine which literatures were finally included. Resolve differences through discussion. Data in the included studies were independently extracted by two investigators, including first author, country or region, publication year, study type, sample size, primary outcomes, main findings, and so on.

Two investigators independently assessed the quality of the included studies. Resolve differences through discussion. The methodological quality of case control or cohort studies was assessed using the Newcastle‐Ottawa Scale (NOS).^18^ The judging criteria of NOS include selection, comparability, and exposure (case‐control study) or outcomes (cohort study). The criteria of an observational study by the Agency for Healthcare Research and Quality (AHRQ) were used to evaluate cross‐sectional studies.^19^ AHRQ evaluation criteria consist of 11 items. The answer of each item is yes, no, or unclear. Only the answer of yes scores 1, and the others score 0. Studies with scores below 6 indicate low quality, while a score of 6 or above indicates good quality.

### 2.4 Statistical analysis

We used stata 14.0 software for data analysis. This study was registered on PROSPERO: CRD42021229728. Weighted mean deviation (WMD) and 95% confidence intervals (CIs) were calculated for each related study. Heterogeneity was assessed by *I* ^2^ test. When *I* ^2^ ≥ 50%, we used the random effect model for meta‐analysis; otherwise, we used the fixed‐effects model. We performed sensitivity analysis to investigate the sources of heterogeneity. There were not enough studies (*n* < 10) to analyze the publication bias.

## 3 RESULTS

### 3.1 Literature search and study characteristics

**Figure 1:**
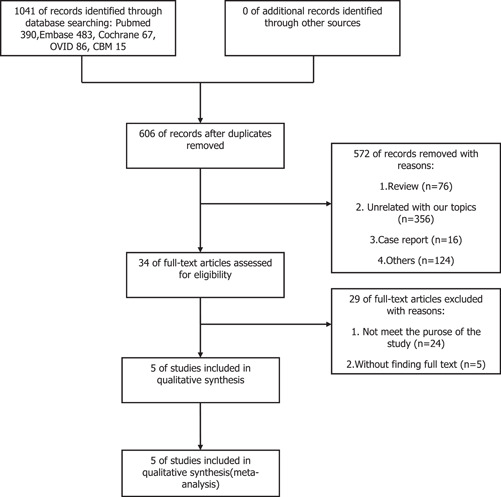
Flowchart of eligible studies

Our initial database search yielded a total of 1041 articles. We screened 606 articles after eliminating duplicates. Then 572 articles were excluded for irrelevance, and the eligibility of 34 full‐text articles was evaluated. Ultimately, we included five studies for systematic review and meta‐analysis after reading the full text. The flow diagram in Figure 1 shows the screening process for included studies.

Among those five studies included, two studies were performed in Italy,^20^, ^21^ two in Spain^22^, ^23^ and the remaining one^24^ in Iran. Two studies^21^, ^24^ were case‐control studies, two^22^, ^23^ were cross‐sectional case‐control studies, one^20^ was retrospective cohort study. Their exclusion criteria indicated that related retinal diseases were excluded in all studies. The quality scores of study design among those five studies included were not less than 6. A total of 401 participants participated in the meta‐analysis. Four^20^, ^21^, ^23^, ^24^ studies included early post‐COVID‐19 patients, one^22^ included patients diagnosed with COVID‐19 (nonacute infectious stage). In three studies,^20^, ^21^, ^22^ OCTA images were obtained using Spectral Domain Zeiss Cirrus 5000‐HD‐OCT Angioplex (Carl Zeiss Meditec, Inc.). One study^23^ obtained OCTA images using the DRI OCT Triton SS‐OCT Angio (Topcon Medical Systems, Inc). The remaining study^24^ performed OCTA scans with the AngioVue (RTVue XR Avanti, Optovue; Software Version 2018.0.0.14) system. Table 1 shows detailed characteristics of studies included.

**Table 1:** Characteristics of included studies

First author	Publication year	Country/area	Study type	Whether to exclude patients with previously diagnosed retinal diseases	Sample size of COVID‐19 patients	Sample size of healthy controls	Ophthalmological examination	Primary outcomes	Main findings	NOS/AHRQ score
Savastano MC	February 2021	Italy	A cohort observational study	Yes	70 (evaluated at 1‐month hospital discharge)	22	OCTA and structural OCT	VD; VP	VD and VP for SCP and DCP showed no significant differences between the groups.	7
Villahoz NG	May 2021	Spain	A cross‐sectional case‐control study	Yes	66 (performed 12 weeks after diagnosis)	29	OCTA	VD; PD	Patients show lower VD and PD compared to controls.	7
Zamora JG	March 2021	Spain	A cross‐sectional, consecutive case‐control study	Yes	25 (14 days after hospital discharge)	25	OCT; OCTA	VD and FAZ area of SCP, DCP and CC	Patients presented significantly thinner GCL and thicker RNFL. In both SCP and DCP, patients presented lower VD in the foveal region and a greater FAZ area.	8
Alfonso Savastano	September 2020	Italy	An observational case‐control study	Yes	80 (1 month from hospital discharge)	30	OCT; OCTA	RPCP‐PD; RPCP‐FI	RPCP‐PD was lower in patients. RNFL average thickness was linearly correlated to RPCP‐FI and RPCP‐PD within post‐COVID‐19 group.	6
Mojtaba Abrishami	February 2021	Iran	A case‐control study	Yes	31 (recovered COVID‐19 patients)	23	OCTA	VD; the area of the FAZ	Mean SCP VD and DCP VD of the COVID cohort were significantly lower in the foveal and parafoveal regions.	6

Abbreviations: CC, choriocapillaris; DCP, deep capillary plexus; FAZ, foveal avascular zone; FI, flow index; GCL, ganglion cell layer; OCTA, optical coherence tomography angiography; PD, perfusion density; RNFL, retinal nerve fiber layer; RPCP, radial peripapillary capillary plexus; SCP, superficial capillary plexus; VD, macular vessel density; VP, vessel perfusion.

### 3.2 Comparison of VD between COVID‐19 or post‐COVID‐19 patients and healthy controls

**Figure 2:**
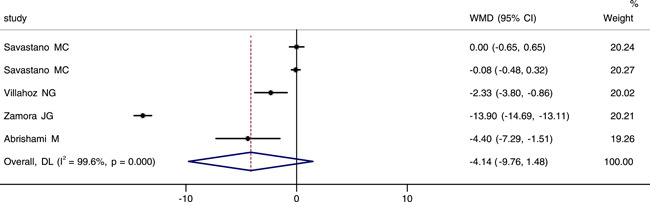
Forest plot of the comparison of foveal vessel density in superficial capillary plexus

Four studies reported and analyzed foveal VD in the superficial capillary plexus (SCP). It was worth noting that in one of those studies, OCTA images were performed with a 3 × 3 and a 6 × 6 mm volume scan pattern centered on the fovea.^20^ We extracted data from these four studies for a meta‐analysis. The integrated results indicated that there was a large heterogeneity among them (*p* < .001, *I* ^2^ = 99.6%), then the random effect model was chosed. The combined results indicated that there was no statistical difference in foveal VD of SCP between groups (WMD = −4.14, 95% CI: −9.76, 1.48), as shown in Figure 2. We performed sensitivity analysis, and the result revealed that no studies changed the overall heterogeneity, which was stable (see Figure 1).

**Figure 3:**
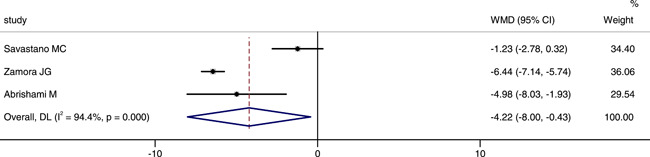
Forest plot of the comparison of foveal vessel density in superficial capillary plexus

**Figure 4:**
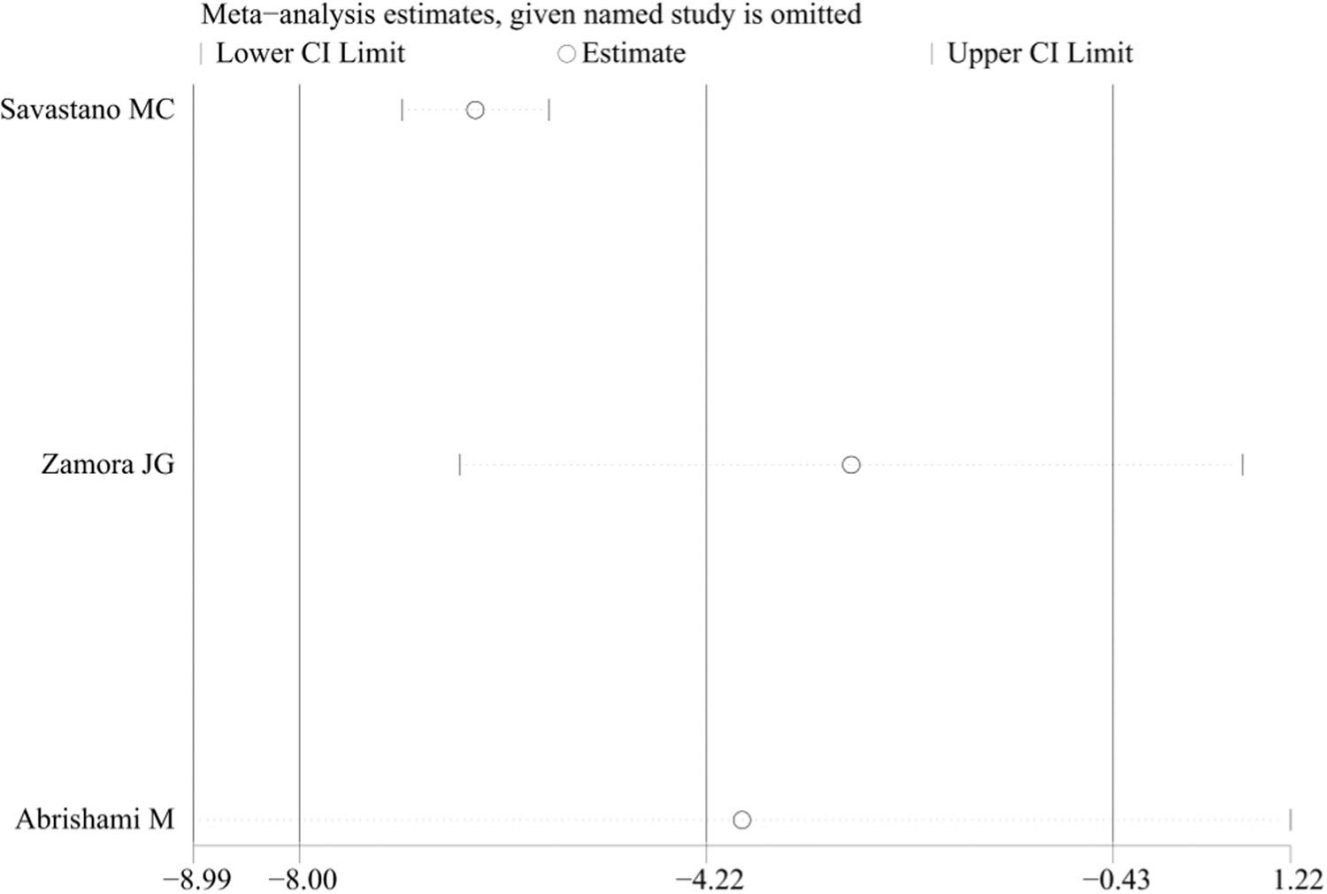
Sensitivity analysis of the comparison of foveal vessel density in superficial capillary plexus

Three studies involved foveal VD in the deep capillary plexus (DCP). We extracted the data from these three studies. The combined results revealed that the foveal VD of DCP in patients was significantly lower than that in control group (WMD = −4.22, 95% CI: −8.00, −0.43), as shown in Figure 3. The random‐effect model was chosed due to a large heterogeneity (*p *< .001, *I* ^2^ = 94.4%). We also conducted sensitivity analysis to investigate the sources of heterogeneity. The result also showed that no studies changed the overall heterogeneity, which was stable, as shown in Figure 4.

Three studies analyzed the parafoveal VD of SCP between the two groups. We extracted data from these studies. The pooled results indicated that there was no significant difference in parafoveal VD of SCP between the two groups (WMD = −5.26, 95% CI: −12.26, 1.75). We chosed the random effect model because of a large heterogeneity (*p* < .001, *I* ^2^ = 99.5%). Sensitivity analysis was also performed and the result was equally stable (see Figures 2 and 3).

**Figure 5:**
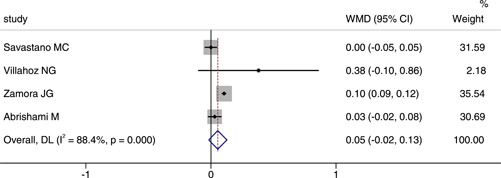
Forest plot of the comparison of the area of foveal avascular zone

Two studies involved the parafoveal VD of DCP between the two groups. The pooled results revealed that there was also no statistical difference between groups (WMD = −1.25, 95% CI: −3.25, 0.76). The random‐effect model was selected because of a large heterogeneity (*p* = .027, *I* ^2^ = 79.5%). Sensitivity analysis was also performed and the result was still equally stable (see Figures 4 and 5).

## 4 COMPARISON OF THE AREA OF FAZ BETWEEN COVID‐19 OR POST‐COVID‐19 PATIENTS AND HEALTHY CONTROLS

**Figure 6:**
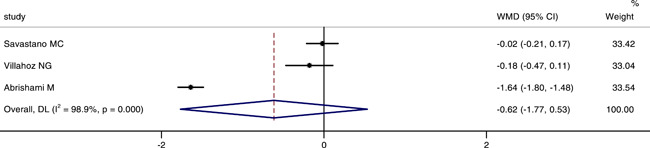
Forest plot of the comparison of the perimeter of foveal avascular zone

Four studies reported the area of FAZ between COVID‐19 or post‐COVID‐19 patients and healthy controls. The comprehensive results showed that WMD was 0.05, 95% CI (−0.02, 0.13), indicating no significant difference between the two groups, as shown in Figure 5. We chosed the random effect model because of a large heterogeneity (*p* < .001, *I* ^2^ = 88.4%). The result of sensitivity analysis was also stable (see Figure 6).

### 4.1 Comparison of the perimeter of FAZ between COVID‐19 or post‐COVID‐19 patients and healthy controls

**Figure 7:**
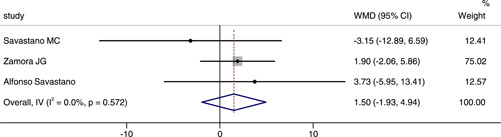
Forest plot of the comparison of central foveal thickness

Three studies involved the perimeter of FAZ between the two groups. The integrated results indicated that there was no significant difference in the perimeter of FAZ between groups (WMD = −0.62, 95% CI: −1.77, 0.53), as shown in Figure 6. The random‐effect model was also chosed, because there was a large heterogeneity (*p* < .001, *I* ^2^ = 98.9%). The result of sensitivity analysis was also stable (see Figure 7).

### 4.2 Comparison of CFT between COVID‐19 or post‐COVID‐19 patients and healthy controls

Three studies reported the CFT between COVID‐19 or post‐COVID‐19 patients and healthy controls. The combined results showed a WMD of 1.50, 95% CI (−1.93, 4.94), which suggested there was no significant difference between groups. There was no statistical heterogeneity between two groups (*p* = .572, *I* ^2^ = 0.0%), and the fixed effect model was chosed (Figure 7).

### 4.3 Comparison of SCT between COVID‐19 or post‐COVID‐19 patients and healthy controls

**Figure 8:**
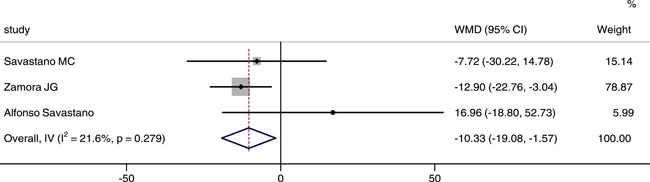
Forest plot of the comparison of subfoveal choroidal thickness

Three studies reported and analyzed SCT between two groups. The integrated results showed that the SCT in patients was significantly thinner than that in control group (WMD = −10.33, 95% CI: −19.08, −1.57), as shown in Figure 8. There was no statistical heterogeneity between groups (*p* = .279, *I* ^2^ = 21.6%), we chose the fixed effect model.

## 5 DISCUSSION

Our meta‐analysis suggests that nonacute infectious COVID‐19 or post‐COVID‐19 patients present significantly lower foveal VD of the DCP and thinner SCT than healthy controls. There is a quantitative difference in retinal microcirculation between the two groups. Our main findings provide information on SARS‐CoV‐2 infection with retinal microvascular involvement. To our knowledge, this is the first meta‐analysis on the quantitative analysis of retinal microvascular involvement in SARS‐CoV‐2 infection.

In these studies we included, the subjects included COVID‐19 and post‐COVID‐19 patients. Ophthalmological examination and OCTA were performed 12 weeks after diagnosis for COVID‐19 patients, and 2 weeks or 1 month after hospital discharge for post‐COVID‐19 patients. These results suggest that the impairment of SARS‐CoV‐2 to retinal microvessels may be a medium to long term process. One study also found that 22% of patients showed retinal microangiopathy manifested as cotton wool spots at average 43 days after COVID‐19 symptoms onset.^25^ The retina is the body's most metabolically demanding tissue. The foveal flow impairment may occur, when thrombosis occurs in blood vessel and blood flow in vascular bed slows down, as observed in our study, VD is decreased. Inflammation and endothelial dysfunction may also be involved in this process. In addition, we also found that the subfoveal choroidal thickness became thinner in COVID‐19 patients. As we all know, choroidal blood flow is abundant, accounting for about 95% of the blood flow in the eyeball, which is the main source of blood supply to the outer retina and macula. The choroidal thickness in macular area can indirectly reflect the choroidal blood flow. Given the presence of ACE2 receptors in choroid, choroidal vessels involvement of SARS‐CoV‐2 may be expected.

The mechanism by which SARS‐CoV‐2 may affect the retinal vascular system has not been unanimously concluded. Some scholars have explained several possible mechanisms. First of all, ACE2, which has been found in eyes in connection with Muller cells, retinal pigment epithelium, and pericytes of endothelial cells, is the main receptor of SARS‐CoV‐2 cellular entry.^26^ Furthermore, a novel SARS‐CoV‐2 entry route has been revealed, CD147‐spike protein, which has been shown a moderate to high expression in human retina.^27^, ^28^ Microvascular endothelial injury and cytokine oversecretion are also thought to be key factors due to their important roles in controlling vascular tension and protecting the blood‐retinal barrier. In addition, The concept of thromboinflammation should not be ignored. SARS‐CoV‐2 can cause direct cytopathy and indirect damage associated with intense inflammatory response and hypercoagulant state it induces.^22^

It has also been suggested that the retinal vascular system may be altered by a conjunction of events including thromboembolism, hypercoagulability, hypoxia, and endothelial dysfunction. Moreover, they also found that the retinal vascular changes were not associated with the clinical severity of the disease, suggesting that COVID‐19‐related microvascular involvement may depend on the patient's procoagulant state rather than the severity of the disease.^29^ Unfortunately, most of subjects in our included studies were post‐COVID‐19 patients, and there was not enough data to allow us to conduct a study on the correlation between retinal microvascular changes and disease severity. Nevertheless, in this meta‐analysis, one^22^ of the included studies found no difference in OCTA parameters within the COVID‐19 group when thrombotic events were taken into account, which may suggest that the retinal microvascular involvement of SARS‐CoV‐2 is not restricted by other levels of thrombotic events. Meanwhile, another study^30^ found that the retinal vein diameters were significantly larger in COVID‐19 patients than that in unexposed subjects, and their size was positively correlated with disease severity. The authors suggest that this may be related to the inflammatory response and/or endothelial damage in COVID‐19. It can be seen that the mechanism of SARS‐COV‐2 affecting the retinal vascular system is complex and requires further study.

There are some limitations in our study. First, It is clear to us that the small number of studies available is the reason for the limited effectiveness of our earlier meta‐analyses. Despite a comprehensive search of multiple databases, only five observational studies fitted the bill, and randomized controlled studies were lacking. Fortunately, we did not include low‐quality studies, and the sensitivity analysis indicated that the results were not affected by individual studies in our meta‐analysis. Of course, with more high‐quality, large‐sample randomized or nonrandomized controlled trials, we could draw more reliable conclusions. Second, these studies included did not involve critically ill patients, and the changes in retinal microvessels cannot be detected in patients with acute SARS‐CoV‐2 infection or severe cases. we were also unable to study the correlation between retinal microvascular changes and disease severity. The selected sample of nonacute infectious COVID‐19 or post‐COVID‐19 patients was not fully representative of the average population of COVID‐19 patients. Third, there was a greater number of COVID‐19 patients with immune diseases, arterial hypertension, diabetes mellitus than healthy controls. These factors may be independently associated with SARS‐CoV‐2 infection and should be considered. Fourth, our five included studies did not all use the same device to obtain OCTA images, nor did they use the same scanning pattern, which could also be a source of heterogeneity.

## 6 CONCLUSION

The retinal vascular system has the unique advantage of being easy to study in vivo compared with the vascular systems of other organs. It is of great significance to study retinal microvascular involvement of SARS‐CoV‐2, because this may represent vascular damage in other organs. Therefore, OCTA becomes a valuable clinical tool. The patients of nonacute infectious COVID‐19 or post‐COVID‐19 presented significantly lower foveal VD of DCP and thinner SCT than healthy controls. The impairment of SARS‐CoV‐2 to retinal microvessels and choroidal vessels maybe a medium to long‐term process. These findings provide relevant information on the retinal microvascular damage of SARS‐CoV‐2, and also indicate that vascular damage may occur in other organs. This has important implications for follow‐up and identification of short‐and long‐term sequelae that may occur in COVID‐19 patients.

## CONFLICTS OF INTEREST

The authors declare no conflicts of interest.

## ETHICS STATEMENT

All analyses in this study are based on published research and do not require ethical approval or informed consent, but remain in full compliance with the Declaration of Helsinki.

## References

[1] Bertoli F , Veritti D , Danese C , et al. Ocular findings in COVID‐19 patients: a review of direct manifestations and indirect effects on the eye. J Ophthalmol. 2020;2020:4827304. 32963819 10.1155/2020/4827304 PMC7491448

[2] Giamarellos‐Bourboulis EJ , Netea MG , Rovina N , et al. Complex immune dysregulation in COVID‑19 patients with severe respiratory failure. Cell Host Microbe. 2020;27(6):992‐1000. e3. 32320677 10.1016/j.chom.2020.04.009 PMC7172841

[3] Lai CC , Ko WC , Lee PI , Jean SS , Hsueh PR . Extra‐respiratory manifestations of COVID‐19. Int J Antimicrob Agents. 2020;56(2):106024. 32450197 10.1016/j.ijantimicag.2020.106024 PMC7243791

[4] Siedlecki J , Brantl V , Schworm B , Mayer WJ , Priglinger S . COVID‐19: ophthalmological aspects of the SARS‐CoV 2 global pandemic. Klin Monbl Augenheilkd. 2020;237:675‐680. 32375197 10.1055/a-1164-9381 PMC7295305

[5] Inomata T , Kitazawa K , Kuno T , et al. Clinical and prodromal ocular symptoms in coronavirus disease: a systematic review and meta‐analysis. Invest Ophthalmol Vis Sci. 2020;61(10):29. 10.1167/iovs.61.10.29 PMC7441339 32797198

[6] Insausti‐García A , Reche‐Sainz JA , Ruiz‐Arranz C , et al. Papillophlebitis in a COVID‐19 patient: inflammation and hypercoagulable state. Eur J Ophthalmol. 2020;32:NP168‐NP172. 32735134 10.1177/1120672120947591 PMC7399568

[7] Yan R , Zhang Y , Li Y , Xia L , Guo Y , Zhou Q . Structural basis for the recognition of SARS‐CoV‐2 by full‐length human ACE2. Science. 2020;367(6485):1444‐1448. 32132184 10.1126/science.abb2762 PMC7164635

[8] Hamming I , Timens W , Bulthuis ML , Lely AT , Navis G , van Goor H . Tissue distribution of ACE2 protein, the functional receptor for SARS coronavirus. A first step in understanding SARS pathogenesis. J Pathol. 2004;203(2):631‐637. 15141377 10.1002/path.1570 PMC7167720

[9] Li MY , Li L , Zhang Y , Wang XS . Expression of the SARS‐CoV‐2 cell receptor gene ACE2 in a wide variety of human tissues. Infect Dis Poverty. 2020;9(1):45. 32345362 10.1186/s40249-020-00662-x PMC7186534

[10] Liu L , Sun Y , Pan X , Shen W . Expression of SARS coronavirus S protein functional receptor—angiotensin‐converting enzyme2 in human cornea and conjunctiva. Ophthalmic Res. 2004;2(6):561‐564.

[11] Senanayake P , Shadrach JDK , Milsted A , et al. Angiotensin II and its receptor subtypes in the human retina. Invest Ophthalmol Vis Sci. 2007;48(7):3301‐3311. 17591902 10.1167/iovs.06-1024

[12] Casagrande M , Fitzek A , Püschel K , et al. Detection of SARS‐CoV‐2 in human retinal biopsies of deceased COVID‐19 patients. Ocul Immunol Inflamm. 2020;28(5):721‐725. 32469258 10.1080/09273948.2020.1770301

[13] Seah I , Agrawal R . Can the coronavirus disease 2019 (COVID‐19) affect the eyes? a review of coronaviruses and ocular implications in humans and animals. Ocul Immunol Inflamm. 2020;28(3):391‐395. 32175797 10.1080/09273948.2020.1738501 PMC7103678

[14] Kasal DA , De Lorenzo A , Tibiriçá E , et al. COVID‐19 and microvascular disease: pathophysiology of SARS‐CoV‐2 infection with focus on the renin‐angiotensin system. Heart Lung Circ. 2020;29(11):1596‐1602. 32972810 10.1016/j.hlc.2020.08.010 PMC7467122

[15] Marinho PM , Marcos AAA , Romano AC , Nascimento H , Belfort R Jr. Retinal findings in patients with COVID‐19. Lancet. 2020;395(10237):1610. 32405105 10.1016/S0140-6736(20)31014-X PMC7217650

[16] Goyal M , Murthy SI , Annum S . Retinal manifestations in patients following COVID‐19 infection: a consecutive case series. Indian J Ophthalmol. 2021;69(5):1275‐1282. 33913876 10.4103/ijo.IJO_403_21 PMC8186578

[17] Kashani AH , Chen CL , Gahm JK , et al. Optical coherence tomography angiography: a comprehensive review of current methods and clinical applications. Prog Retin Eye Res. 2017;60:66‐100. 28760677 10.1016/j.preteyeres.2017.07.002 PMC5600872

[18] Wells GA , Shea B , O'Connell D . The Newcastle‐Ottawa Scale (NOS) for assessing the quality of non‐randomised studies in meta‐analysis. Ottawa Health Research Institute; 2004.

[19] Rostom A , Dubé C , Cranney A . Celiac disease. Evidence Reports/Technology Assessments Agency for Healthcare Research and Quality; 2004.

[20] Savastano MC , Gambini G , Cozzupoli GM , et al. Retinal capillary involvement in early post‐COVID‐19 patients: a healthy controlled study. Graefes Arch Clin Exp Ophthalmol. 2021;1:1‐9. 10.1007/s00417-020-05070-3 PMC7848665 33523252

[21] Savastano A , Crincoli E , Savastano MC , et al. Peripapillary retinal vascular involvement in early post‐COVID‐19 patients. J Clin Med. 2020;9(9):2895. 10.3390/jcm9092895 PMC7565672 32911619

[22] Guemes‐Villahoz N , Burgos‐Blasco B , Vidal‐Villegas B , et al. Reduced macular vessel density in COVID‐19 patients with and without associated thrombotic events using optical coherence tomography angiography. Graefes Arch Clin Exp Ophthalmol. 2021;7:1‐7. 10.1007/s00417-021-05186-0 PMC8102150 33961108

[23] González‐Zamora J , Bilbao‐Malavé V , Gándara E , et al. Retinal microvascular impairment in COVID‐19 bilateral pneumonia assessed by optical coherence tomography angiography. Biomedicines. 2021;9(3):247. 33801324 10.3390/biomedicines9030247 PMC7998142

[24] Abrishami M , Emamverdian Z , Shoeibi N , et al. Optical coherence tomography angiography analysis of the retina in patients recovered from COVID‐ 19: a case‐control study. Can J Ophthalmol. 2021;56(1):24‐30. 33249111 10.1016/j.jcjo.2020.11.006 PMC7666612

[25] Landecho MF , Yuste JR , Gándara E , et al. COVID‐19 retinal microangiopathy as an in vivo biomarker of systemic vascular disease? J Intern Med. 2021;289(1):116‐120. 32729633 10.1111/joim.13156

[26] Zhu P , Verma A , Prasad T , Li Q . Expression and function of Mas‐related G protein‐coupled receptor D and its ligand alamandine in retina. Mol Neurobiol. 2020;57(1):513‐527. 31392515 10.1007/s12035-019-01716-4

[27] Wang K , Chen W , Zhang Z , et al. CD147‐spike protein is a novel route for SARS‐CoV‐2 infection to host cells. Signal Transduct Target Ther. 2020;5(1):283. 33277466 10.1038/s41392-020-00426-x PMC7714896

[28] Hamashima K , Gautam P , Lau KA , et al. Potential modes of COVID‐19 transmission from human eye revealed by single‐cell atlas. BioRxiv 10.1101/2020.05.09.085613

[29] Guemes‐Villahoz N , Burgos‐Blasco B , Vidal‐Villegas B , et al. Reduced retinal vessel density in COVID‐19 patients and elevated D‐dimer levels during the acute phase of the infection. Med Clin (Engl Ed). 2021;156(11):541‐546. 34109275 10.1016/j.medcle.2020.12.020 PMC8178962

[30] Invernizzi A , Torre A , Parrulli S , et al. Retinal findings in patients with COVID‐19: results from the SERPICO‐19 study. EClinicalMedicine. 2020;27:100550. 32984785 10.1016/j.eclinm.2020.100550 PMC7502280

